# Treadmill Hand Injuries Among Children: A Retrospective Case Series From Hospitalized Patients

**DOI:** 10.3389/fped.2021.633091

**Published:** 2021-02-17

**Authors:** Yunxuan Zhang, Yan Liu, Xingang Yuan, Jun Xiao, Xionghui Ding, Qiang Chen, Lin Qiu

**Affiliations:** ^1^Burn and Plastic Surgery Department, Children's Hospital of Chongqing Medical University, Chongqing, China; ^2^Ministry of Education Key Laboratory of Child Development and Disorders, National Clinical Research Center for Child Health and Disorders, China International Science and Technology Cooperation Base of Child Development and Critical Disorders, Chongqing, China; ^3^Chongqing Key Laboratory of Pediatrics, Chongqing, China

**Keywords:** hand injury, pediatric/child, treadmill, epidemiology, treatment

## Abstract

**Background:** With the progress of modernization, treadmill hand injury in pediatric population is taking on a global trend in recent years. The purpose of this study was to investigate the epidemiology and clinical features in a developing country, thereby providing some experience in the treatment and prevention of this particular type of injury.

**Methods:** A 5-year retrospective review of patients with treadmill hand injury in Burn and Plastic Surgery ward at Children' Hospital of Chongqing Medical University was conducted. Demographics, injury details, therapy performed, length of hospital stay, complications, and outcome were analyzed.

**Results:** Forty-six patients were surveyed, with a mean age of 3.5 ± 2.0 years old, including 24 males and 22 females. Injuries (77.8%) occurred between dinner to bedtime, and 95.7% happened indoors. Fingers were the most vulnerable part, of which the middle finger, ring finger, and index finger were the top three ones. The mean body surface area (BSA%) was 0.3 ± 0.2, but at least in deep dermal. Dressing changes, full-thickness skin grafts (FTSG), and Negative Pleasure Wound Therapy (NPWT) assisted FTSG were performed. The scar contracture, as the most severe complication, occurred in 26 patients, of which 22 originally received dressing changes at the time of injury.

**Conclusion:** Treadmill hand injury in children should be highly regarded. Compared with conservative dressing changes, surgical intervention from a professional team may achieve more satisfactory prognosis and fewer complications. A prevention strategy based on “Time-Space-Person” was summarized according to its epidemiological characteristics, may help to decrease the incidence of this specific type of injury theoretically.

## Introduction

The improvement of health consciousness in China society has increased the popularity of exercise equipment, especially the treadmill. A treadmill, with a rubberized belt driven by an electromotor, can cause harm to children by hand contact (1). Usually, curiosity drives children to contact the treadmill with their hands. The lack of self-protection awareness and slow withdraw reflex (2) make hands the most vulnerable parts in children. Treadmill injuries were initially reported by Qatar researcher Attala et al. (3) in 1991, and were subsequently reported in other developed countries, including the United States, the United Kingdom, South Korea, Australia, etc. In recent years, we have also observed an increase of pediatric treadmill hand injuries in Chongqing, a city in the southeast of China. It seems that this type of injury is taking on a global trend.

To the best of our knowledge, this is the first related study from a developing country. In this study, we reported on hospitalized patients who sustained severe hand injuries caused by treadmill contact. The study aimed to investigate the epidemiology and clinical features of this type of injury. The treatments and prevention strategies were emphatically discussed to provide experience and suggestions, thereby promoting global awareness of pediatric treadmill hand injury.

## Methods

### Research Subjects

A single-center retrospective review was conducted for pediatric patients with treadmill hand injuries who were admitted to the Burn and Plastic Surgery ward at Children' Hospital of Chongqing Medical University from January 1, 2015 to December 31, 2019. The guidelines of the Code of Ethics of the World Medical Association were completely fulfilled (Declaration of Helsinki). Ethics approval was granted by Hospital Ethics Committee of Children' Hospital of Chongqing Medical University.

### Inclusion Criteria and Exclusion Criteria

Inclusion criteria were the following: (1) children aged 0–18 years old; (2) inpatients with treadmill hand injuries or other treadmill-related hand diseases; and (3) both the children and their guardians consented to participate in the survey. Exclusion criteria were as following: (1) patients who were transferred to other hospitals <24 h after admission; and (2) outpatients.

### Indicators

Patients were classified into three groups, as follows: (1) the “trauma” group patients who were hospitalized for original wound treatment; (2) the “early complication” group patients who complained of early complications after being treated conservatively; and (3) the “scar contracture” group, patients who complained of scar contracture several months after wound healing. Each case was documented in terms of demographics such as age, gender, time of injury, adult supervision. Each wound was assessed for symptoms, anatomic location, scope and depth, and mechanism of injury. The management received, length of hospital stay (LOS), outpatient follow-up, and complications were also recorded. The outcome assessment was evaluated by parents subjectively, including range of motion (ROM), functional recovery (regarding daily activities), and aesthetic appearance.

### Statistical Analysis

Data were expressed as mean ± standard deviation (SD), percentage with number of patients, and median with the range in parentheses. Comparisons between groups of continuous variables (such as age in gender and prognostic score in treatments) that were normally distributed were carried out using Student's *t*-test or a corrected *t-*test. Comparisons of categorical variables (such as incidence of left and right hand) were performed using the χ^2^-test or Fisher's exact test. Data were analyzed by using SPSS v26.0. *P* values < 0.05 were considered significant.

## Results

During the 5-year study period, 46 patients were admitted, including 24 males and 22 females (Table 1). Thirty-eight (82.6%) patients were from urban areas, eight (17.4%) came from rural areas. The mean age at the time of injury was 3.5 ± 2.0 years old (3.0, 1.3–10.3 years old). Males were statistically older than females (*P* < 0.05). Children aged between 1 and 3 years old accounted for 78.3% of the injuries, whereas the remaining 21.7% were older patients. The incidence increased almost every year, with 16 cases occurring in 2019 so far.

**Table 1:** Patients' demographic and injury data.

**Indicators**	**Mean (Median, Range)**
No. of patients	46
Residence (urban: rural)	38:8 (82.6%:17.4%)
Sex (male: female)	24:22 (52.2%:47.8%)
Mean age (year)	3.5 ± 2.0 (3.0,1.3–10.3)
Male	4.2 ± 2.4 (3.4, 1.4–10.3)
Female	2.8 ± 1.2 (2.7, 1.3–6.6)
**Year of injury**	
2015	6
2016	7
2017	6
2018	11
2019	16

*Data are expressed as mean ± standard deviation (SD), as well as median with range in the parenthesis*.

**Figure 1:**
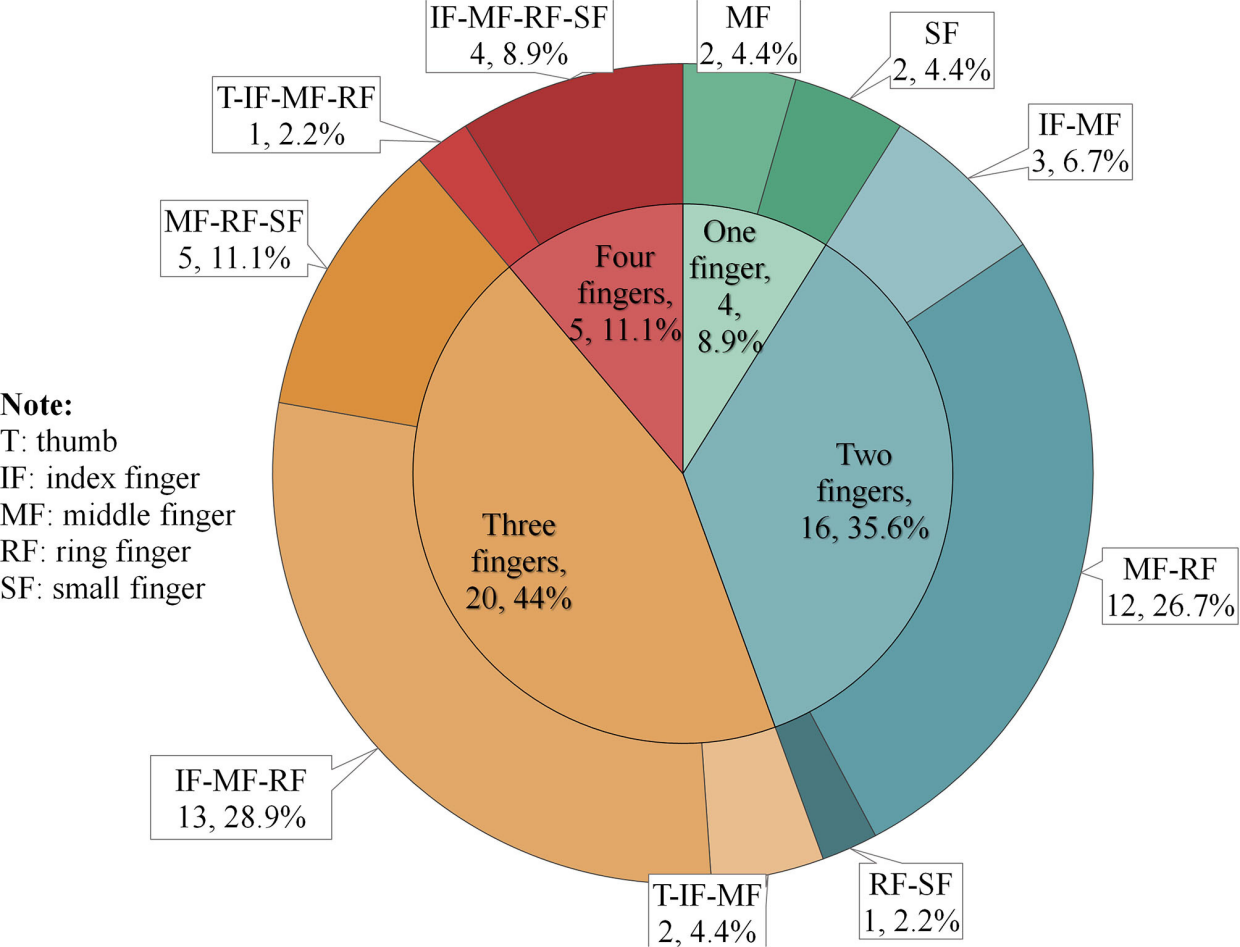
Injury patterns of the involved 116 fingers. The inner circle of the pie chart represents the number of involved fingers in each injured hand. The outer circle of the pie chart shows how these finger injuries are combined.

A total of 48 injured hands were involved, including six opisthenars, six palms, and 116 fingers (Table 2). The right side (*n* = 29) was statistically more vulnerable than the left (*n* = 19) (*P* < 0.05). Of the finger injuries, 42 (36.2%) involved the middle finger, 36 (31.0%) involved the ring finger, and 23 (19.8%) involved the index finger. Multi-finger injury accounted for 91.8% of the injured hands. The most common injury pattern was the simultaneous involvement of index-middle-ring fingers (28.9%) followed by middle-ring fingers (26.7%), as shown in Figure 1.

**Table 2:** Injury details of the involved 48 hands.

**Indicators**	**Total (%)**
**Injury laterality**	48 (100%)
Left	17 (35.4%)
Right	27 (56.3%)
Bilateral (pairs)	2 (8.3%)
**Injury location**	
Palm only	2
Opisthenar only	1
Finger only	36
Palm and finger	4
Opisthenar and finger	5
**Fingers**	
Thumb	3 (2.6%)
Index	23 (19.8%)
Middle	42 (36.2%)
Ring	36 (31.0%)
Little	12 (10.4%)
**Mean BSA, %**	0.3 ± 0.2 (0.2, 0.1–0.7)
**Wound depth**	48 (100%)
Deep dermal	5 (10.4%)
Full thickness	18 (37.5%)
Tendon	3 (6.3%)
Articular capsule	3 (6.3%)
Unspecified^*^	19 (39.6%)

* *Data in “scar contracture” group was unavailable, but due to the scar formation and contracture, we can infer that these depths are at least in deep dermal*.

The mean BSA% was 0.3 ± 0.2 and ranged from 0.1 to 0.7. In terms of depth, five (10.4%) sustained deep dermal area, 18 (37.5%) reached full-thickness, three (6.3%) injured tendons, and three (6.3%) involved the interphalangeal joint level. Considering the length of time that passed since the injury occurred in the “scar contracture” group, scope, and depth data were unavailable; however, we could infer that these wounds were in the deep dermal region (39.6%).

Twenty patients were identified in the “trauma” group and the mean time from injury to admission was 13.2 ± 23.8 h (4.0, 1.0–96.0 h). If we excluded the three patients who were admitted after 1 day, then the result was 4.2 ± 2.9 h. They all complained of soft tissue injuries, including abrasions and contusions, or skin laceration of the hand region. Seventeen cases (85.0%) were recorded as “slight bleeding,” two cases (10.0%) were “severe bleeding,” 14 (70.0%) had wounds with irregular edge, two (10.0%) had regular edge, and in five cases, the wound was contaminated by oil (25.0%).

**Figure 2:**
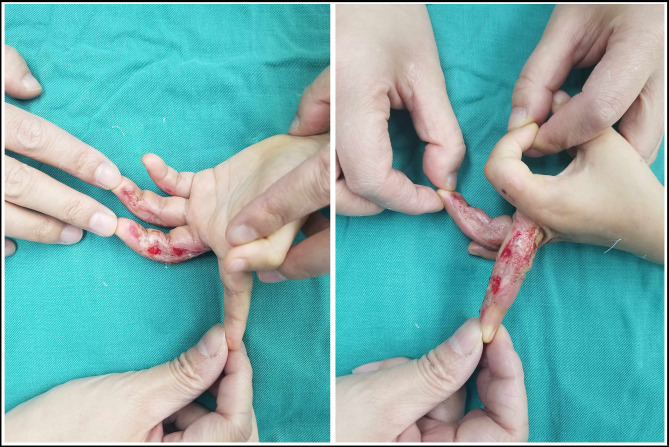
Treadmill hand injury in the index finger, middle finger, and ring finger of the right hand. The patient initially received 9 days of outpatient dressing changes in our center. The wound in index finger and small finger were almost healed. However, the wound in middle finger and ring finger were serious, presenting a poor healing.

Seven patients were identified in the “early complication” group. They received several days of outpatient dressing changes, and the mean time from injury to admission (to our center) was 12.3 ± 8.5 days (11.0, 3.0–30.0 days). Four patients (57.1%) complained of increased local secretions (suspected infection), and the other three (42.9%) were due to wound healing difficulties (Figure 2). These early complications were accompanied by non-healing wounds, leading to an urgent need for hospitalization for further treatment. Therefore, managements were almost the same as those in the “acute trauma” group.

These patients (*n* = 27) all received occupational wound care and/or surgical intervention in our ward, including six cases of dressing changes, eight cases of full-thickness skin grafts (FTSG), and 13 cases of Negative Pressure Wound Therapy (NPWT) assisted FTSG. The mean LOS in patients who received dressing changes in our center was 9.8 ± 6.7 days, while the result in surgical intervention group was 17.0 ± 9.7 days. Elastic bands and silicone were recommended for functional recovery and scar prevention. Those who received dressing changes had a statistically lower score in ROM than those underwent surgical intervention (*P* < 0.05). No statistically significant difference was found in the functional recovery and appearance between the two groups.

Nineteen patients were identified in the “scar contracture” group. These patients came to our center for the first time and were hospitalized for scar contracture after wound healing. In addition, these patients were initially treated conservatively in local clinics or emergency departments, and then received dressing changes at community clinics or by themselves. Auxiliary equipment, such as elastic bands and static splints, were also not recommended. The mean time from injury to admission was 10.3 ± 7.1 months (8.0, 4.0–36.0 months).

**Figure 3:**
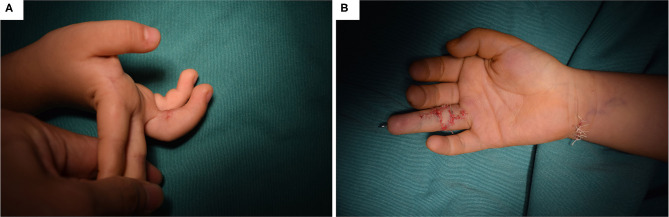
Scar contracture in a rain finger and its corresponding management. **(A)** The scar contracture occurred in the proximal interphalangeal joint of the ring finger, with 45 degrees. **(B)** The Z-plasty scar release and FTSG, as well as the Kirschner wire internal fixation.

The late complications in this study included pigmentation and scar contracture. Although some parents complained of pigmentation more or less during follow-up, they all accepted the results. However, scar contracture was a severe complication resulting in loss of range of motion in 26 patients (Table 3). These scars all occurred on the volar sides of the finger and involved four metacarpophalangeal joints (MPJ), 34 proximal interphalangeal joints (PIPJ), 38 distal interphalangeal joints (DIPJ), as well as four web spaces. The mean degree of contraction was 36.0 ± 20.0 degrees. Twenty patients received the Z-plasty scar release and FTSG, among which six additionally underwent internal fixation with Kirschner wire (Figure 3). The mean LOS was 7.3 ± 1.0 days, and the Kirschner wires were removed 2–3 weeks after discharge. Finally, the scar release was successful in 90% of the patients, who subsequently demonstrated a full range of motion of the affected fingers. One patient was still reluctant to use the affected finger, and another was unsatisfied with the appearance. The other six patients were still waiting for their appointments.

**Table 3:** The prognosis of different managements, as well as further treatments of the complication (scar contracture).

	**Managements**		
	Dressing Change^*^	**Surgical Intervention**	Dressing Change^**^
**Number of Patients**	6	21	19
LOS^&^ (day)	9.8 ± 6.7 (7.5, 5–23)	17.0 ± 9.7 (18, 4–40)	NA
Mean follow-up (month)	9.9 ± 2.8 (9.0, 6.0–16.0)	NA	
**Outcome^#^**			
ROM	8.0 ± 1.1 (8.0, 7.0–9.0)	9.1 ± 1.1 (9.0, 7.0–10.0)	NA
Function	9.5 ± 0.8 (10.0, 8.0–10.0)	9.6 ± 0.6 (10.0, 8.0–10.0)	NA
Appearance	8.5 ± 0.5 (8.5, 8.0–9.0)	8.8 ± 0.5 (9.0, 8.0–9.5)	NA
**Scar contracture**	3	4	19
**Location of contracture**	MJ × 4, PIJ × 34, DIJ × 38, web space × 4		
**Degree of contracture**	36.0 ± 20.0 (30.0, 15.0–90.0)		
**Treatment**	Scar release and FTSG: 14		
	Scar release and FTSG with Kirschner wire internal fixation:6		
**LOS**^&&^ (day)	7.3 ± 1.0 (7.0, 5.0–9.0)		

*Data are expressed as mean ± standard deviation (SD), as well as median with range in the parenthesis*.

*FTSG, full-thickness skin graft; NPWT, Negative Pressure Wound Therapy; LOS, length of hospital stay; ROM, range of motion; MPJ, metacarpophalangeal joint; PIPJ, proximal interphalangeal joint; DIPJ, distal interphalangeal joint; NA, not applicable*.

* *Patients who received dressing changes in our center*.

** *Patients who received dressing changes in local hospital and complained of scar contracture*.

& *The length of hospital stay of initial management*.

&& *The length of hospital stay of scar release procedure*.

# *Outcomes were evaluated subjectively by parents, with each score of 10 out of 10*.

**Figure 4:**
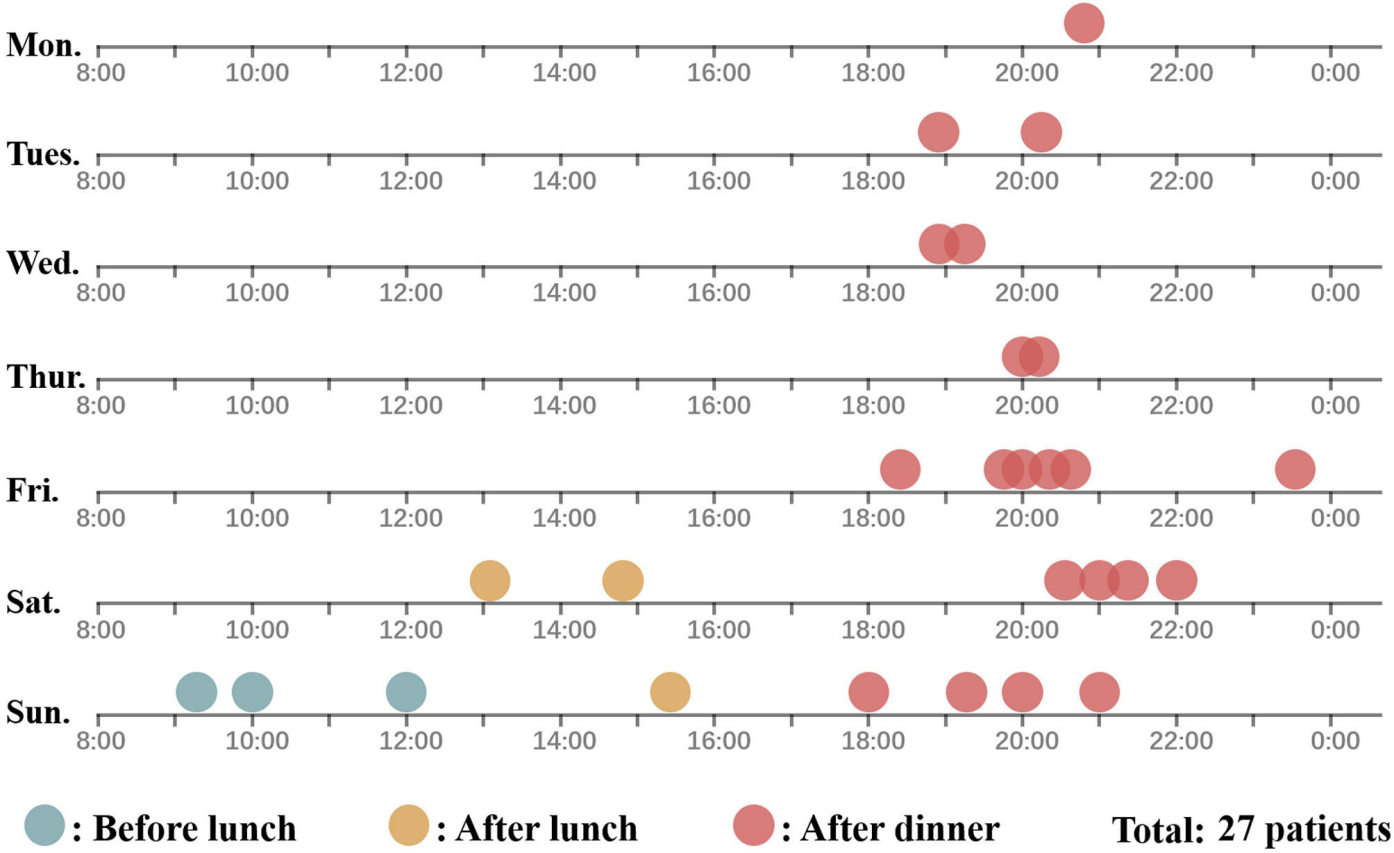
The time of injury in 27 patients according to the day of the week and the time of the day. The time of injury in “trauma group” and “early complication group” (27 patients) are depicted in this figure. A dot represented a patient. Blue, yellow, and red represent the injury that occurred before lunch, between lunch and dinner, and after dinner. Data in “scar contracture” group were not included because the first-hand data were unavailable, and their parents failed to recall the exact time of injury.

By consulting the medical records, we found that 74.1% of injuries occurred during Friday–Sunday. Additionally, the great majority of patients (77.8%) were injured during the period from dinner to bedtime (Figure 4). Data in “scar contracture” group were not included, because the first-hand data were unavailable and their parents failed to recall the exact time of injury. Forty-four cases (95.7%) were injured at home, and two (4.3%) were at the public gym. The most common mechanism of injury was the finger sticking between the moving belt and the base (93.5%). Three (6.5%) cases had their hands directly in contact with the surface of the moving belt. In terms of the surroundings, 37 cases (80.4%) happened when an adult was using the treadmill and the child approached unnoticed from behind. The other nine patients (9.6%) were injured while using the treadmill alone or under supervision by their peers.

## Discussion

The treadmill is usually safe for adults, but it is a potential threat for children that could be easily neglected. In the past few years, we found a rising incidence in pediatric treadmill hand injuries, thus warranting an increased awareness. In this study, we retrospectively reviewed the medical records of hospitalized patients with treadmill hand injuries.

The numbers of male and female patients in this study were roughly equal. Children younger than 4 years old accounted for the great majority (78.3%), which was basically consistent with previous studies (4, 5). Children of this age have significantly high exposure to the outside world after learning to walk and run, while their insufficient awareness of the dangers ultimately leads to the injury.

Despite the limited number of cases, we have summarized some characteristics of this type of injury. A higher incidence was observed in the right hand than the left, possibly due to the fact that the dominant hand usually plays the role of exploration and protection. Fingers were much more vulnerable than the palm or the opisthenar. Multi-finger injuries accounted for the great majority of the injured hands, which made middle finger (36.2%), ring finger (31.0%), and index finger (19.8%) the top three vulnerable fingers. These three fingers are at the distal end of the limb, and thus, they could be easily caught between the belt and the base. Another notable characteristic was that the wound often manifested as “slight bleeding” (85.0%) with small size (<1% TBSA), which could be an important reason for such injuries were not valued in general emergency department. However, these wounds were usually in deep dermis at least. If treated inappropriately, healing difficulty and scar contracture may happen.

In terms of treatment, although the treadmill hand injuries are not intractable, occupational wound management in a specialized ward is necessary. Auxiliary measures, such as static splints, elastic bands, and guidelines for functional recovery, are emphasized by specialists. In contrast, this type of injury with a relatively small size and slight bleeding may not be valued in the emergency department. Moreover, a long-term follow-up from a specialist is extremely necessary. There were three patients developed minor scar contractures 2 months after discharge. Through telephone follow-up, we recommended nighty splinting and passive stretching, which finally helped the patient achieve full ROM recovery.

Besides, the initial management seems to be related to the prognosis of wound healing (1, 6), and conservative managements has been recommended in previous studies to exclude patients who do not require surgical intervention (7–9), while conservative management could lead to deterioration in some large and deep wounds, the proof of which is the occurrence of wound healing difficulties and/or infections after conservative treatment in seven cases. Active surgical intervention was associated with better prognosis in ROM and fewer complications (scar contracture). Re-admission to the hospital for scar release forces patients to re-experience the pain of their injury and to undergo a prolonged recovery process, which can cause functional sequela and psychological problems (10).

In our practice, NPWT assisted FTSG was applied in the most severe cases. The NPWT is an effective device to wound treatment, particularly those in the hand (11, 12). It maintains an enclosed and moist environment, which can promote primary healing and preparation for wound bed before tissue coverage (13). Additionally, the negative pressure suction ensures that the wound is in a non-static environment, which can remove contaminants and protect new granulation tissues. Although there is no consensus on the efficacy of NPWT in inhibiting wound infections (14, 15), this device seems to improve scar appearance (16). For the setting of negative pressure at different ages, we applied persistent suction of −50 to −75 mmHg for children younger than 2 years old, and −75 to −100 mmHg for children older than 2 years old (13). During 7–10 days of NPWT placement, static splinting was utilized to stretch the finger. Dressing changes were not required in this time period, and patients did not suffer from physical or mental agony, thereby reducing the workload of medical staff. The procedure of final FTSG ultimately depended on the granulation tissue formation. If the blood circulation was not adequate, then multistage NPWT might be required.

There's no doubt that, ultimately, whether conservative or aggressive managements is performed, the key point is prevention. The current efforts to prevent treadmill hand injury in children are not adequate. Several studies have introduced various modifications in the treadmill machine itself, including the additional shield, childproof switches, and a belt that stops easily (17–20), etc. On this basis, we believe that a prevention strategy based on “Time-Space-Person” is extremely necessary. In terms of “Time,” these injuries mostly occurred from Friday to Sunday, and from dinner to bedtime. Social factors are possibly related to the results that adults may get their leisure time and exercise with a treadmill in these periods in China. In this aspect, we recommend publishing social media tips, such as public advertisements on TV and push service of mobile phones, during these time periods to remind parents of this type of injury.

In terms of “Space,” the home is the main battlefield for prevention. Unlike the outside road where parents are more vigilant and pay great attention to their children's surroundings, the home is an extremely relaxed environment, so children's behaviors could be neglected, such as contacting the running treadmill from behind. Some researchers suggested that the treadmill should be kept in a locked room designed for exercise (7). Wong et al. (17) suggested placing a back mirror, which might be a practical solution for adults to enjoy exercise without ignoring the child's approach. On the other hand, two cases of injuries that happened in public gyms in this study were from rural areas, which may not be a coincidence. Lack of management experience in exercise equipment, the public gyms in rural areas seems to increase the morbidity of the injury. Therefore, gym managers should understand the potential threats of these machines and rigorously restrict participants accordingly.

In terms of “Person,” 80.4% of the injuries occurred when an adult was using the treadmill. It seems that parents' behavior and the injury are closely related. After all, most young children may not have the ability to turn on the treadmill alone. It was worth noticing that two cases in this study were directly caused by a peer friend suddenly increasing the speed of the treadmill. Therefore, risk factors are not limited to the machine itself, but also the present of parents and children's peer friends. Parents should be cautious with their surroundings and avoid wearing headsets (18) or other devices that interfere with perception when using the treadmill, and children should be banned from using treadmills alone or with their friends.

Prevention strategies at other levels also need to be strengthened, including public education in relevant places (1) and the legislation of displaying warnings (20). Furthermore, we propose that a risk consent form is necessary when selling a treadmill.

As a retrospective single-institution review, this study has several limitations. Even though our institution is the largest child burn center in southwest China, we can only provide local experience and limited cases. The prognosis was evaluated subjectively, which could generate inevitable biases. Comprehensive and multicenter studies are therefore needed to illustrate the accurate incidence and to provide guidelines for clinical management.

## Conclusions

The findings of this study illustrate that treadmill hand injury in children presents a rising trend. The wound is usually small in size with slight bleeding, but can easily involve multiple fingers and deep dermal level. Surgical intervention (FTSG/ NPWT-assisted FTSG) may achieve fewer complications and better prognosis than conservative management. Auxiliary measures, such as static splints, passive stretching, and close follow-up, should also be emphasized. To address the problem at its source, a prevention strategy based on “Time-Space-Person” is extremely necessary:

(1) “Time:” Social media tips via public advertisements on TV and push service of mobile phones during high-risk time period may help remind parents of the potential hazards of the treadmill.
(2) “Space:” The family treadmill should be locked in an exercise room with back mirrors and should be carefully used in an enclosed environment without children. Public gym managers should rigorously restrict participants, especially in rural areas.
(3) “Person:” Parents should regulate their habits of using the treadmill to play an exemplary role. Children should be banned from using a treadmill alone or with their peer friends.

## Data Availability Statement

The raw data supporting the conclusions of this article will be made available by the authors, without undue reservation.

## Ethics Statement

The studies involving human participants were reviewed and approved by Hospital Ethics Committee of Children' Hospital of Chongqing Medical University. Written informed consent to participate in this study was provided by the participants' legal guardian/next of kin.

## Author Contributions

LQ, YL, and YZ conceptualized and designed the study. LQ and XY provided administrative support. JX and XD provided the study materials or patients. YZ was in-charge of the collection and assembly of the data. YZ, JX, and XD analyzed and interpreted the data. All authors wrote and gave the final approval of the manuscript and approved the submitted version.

## Conflict of Interest

The authors declare that the research was conducted in the absence of any commercial or financial relationships that could be construed as a potential conflict of interest.
